# Economic evaluations of radioembolization with yttrium-90 microspheres in liver metastases of colorectal cancer: a systematic review

**DOI:** 10.1186/s12876-023-02793-5

**Published:** 2023-05-24

**Authors:** JC Alonso, I Casans, FM González, D Fuster, A Rodríguez, N Sánchez, I Oyagüez, AO Williams, N Espinoza

**Affiliations:** 1 Nuclear Medicine Department, Hospital Gregorio Marañón, Madrid, Spain; 2 Nuclear Medicine Department, Hospital Clínico Universitario, Valencia, Spain; 3 Nuclear Medicine Department, Hospital Universitario Central, Asturias, Spain; 4 Nuclear Medicine Department, Hospital Clinic, Barcelona, Spain; 5 Nuclear Medicine Department, Hospital Virgen de las Nieves, Granada, Spain; 6 Pharmacoeconomics & Outcomes Research Iberia (PORIB), Madrid, Spain; 7 Boston Scientific Marlborough, Marlborough, MA USA

**Keywords:** Colorectal cancer, Cost, Radioisotope therapy, Systematic review, Yttrium-90

## Abstract

**Background:**

Transarterial radioembolization with yttrium-90 (Y-90 TARE) microspheres therapy has demonstrated positive clinical benefits for the treatment of liver metastases from colorectal cancer (lmCRC). This study aims to conduct a systematic review of the available economic evaluations of Y-90 TARE for lmCRC.

**Methods:**

English and Spanish publications were identified from PubMed, Embase, Cochrane, MEDES health technology assessment agencies, and scientific congress databases published up to May 2021. The inclusion criteria considered only economic evaluations; thus, other types of studies were excluded. Purchasing-power-parity exchange rates for the year 2020 ($US PPP) were applied for cost harmonisation.

**Results:**

From 423 records screened, seven economic evaluations (2 cost-analyses [CA] and 5 cost-utility-analyses [CUA]) were included (6 European and 1 USA). All included studies (n = 7) were evaluated from a payer and the social perspective (n = 1). Included studies evaluated patients with unresectable liver-predominant metastases of CRC, refractory to chemotherapy (n = 6), or chemotherapy-naïve (n = 1). Y-90 TARE was compared to best supportive care (BSC) (n = 4), an association of folinic acid, fluorouracil and oxaliplatin (FOLFOX) (n = 1), and hepatic artery infusion (HAI) (n = 2). Y-90 TARE increased life-years gained (LYG) versus BSC (1.12 and 1.35 LYG) and versus HAI (0.37 LYG). Y-90 TARE increased the quality-adjusted-life-year (QALY) versus BSC (0.81 and 0.83 QALY) and versus HAI (0.35 QALY). When considering a lifetime horizon, Y-90 TARE reported incremental cost compared to BSC (range 19,225 to 25,320 $US PPP) and versus HAI (14,307 $US PPP). Y-90 TARE reported incremental cost-utility ratios (ICURs) between 23,875 $US PPP/QALY to 31,185 $US PPP/QALY. The probability of Y-90 TARE being cost-effective at £ 30,000/QALY threshold was between 56% and 57%.

**Conclusions:**

Our review highlights that Y-90 TARE could be a cost-effective therapy either as a monotherapy or when combined with systemic therapy for treating ImCRC. However, despite the current clinical evidence on Y-90 TARE in the treatment of ImCRC, the global economic evaluation reported for Y-90 TARE in ImCRC is limited (n = 7), therefore, we recommend future economic evaluations on Y-90 TARE versus alternative options in treating ImCRC from the societal perspective.

**Supplementary Information:**

The online version contains supplementary material available at 10.1186/s12876-023-02793-5.

## Background

Colorectal cancer (CRC) is the neoplasm with the highest incidence in Spain and the second-leading cause of cancer death worldwide [1]. It is estimated that 50–60% of patients with CRC develop colorectal metastases [2]. Furthermore, the economic burden of CRC is high with the total cost for metastatic and non-metastatic CRC in Spain in 2012 totalling 986 million euros (€). A major cost component for non-metastatic CRC and metastatic CRC were hospitalization for surgery (86% of the total cost) and non-surgical hospitalization (47% of the total cost), respectively [3].

Clinical practice guidelines such as the American Society of Clinical Oncology (ASCO), the European Society for Medical Oncology (ESMO), and the National Comprehensive Cancer Network (NCCN) recommend surgical resection as a potentially curative first-line treatment for patients with liver metastases from CRC (lmCRC) [4–6]. However, surgical therapy is a feasible option for 10–20% of patients [5], as such the first-line therapy for unresectable lmCRC remains systemic therapy. The following approach is a multidisciplinary therapeutic strategy that, in addition to systemic chemotherapy, includes liver direct therapies such as hepatic arterial infusion, transarterial chemoembolization, and radioembolization with yttrium 90 (Y-90 TARE), whose objective is to facilitate surgical resectability or disease control [5]. Y-90 TARE therapy, either as a monotherapy or combined with systemic therapy, is effective in reducing tumour burden and increasing progression-free and hepatic progression free survival in patients with lmCRC refractory to chemotherapy [7–10]. The ESMO [4], ASCO [5], and NCCN [6] guidelines recommended Y-90 TARE in combination with systemic therapy for lmCRC patients with hepatic predominance metastases and chemotherapy-resistant/-refractory disease. Additionally, the ASCO guideline includes it from second-line setting onwards [5]. Two types of Y-90 microspheres have been evaluated in the treatment of hepatic metastases of CRC: glass (TheraSphere®) [11] and resin (SIR-Spheres®) [12]. Holmium-166 (QuiremSpheres®) [13], a third type of microsphere, has limited clinical evidence for the treatment of lmCRC [14].

Economic evaluations of Y-90 TARE therapy in ImCRC can offer insights to decision-makers on prioritizing health interventions to produce maximum health benefits and financial sustainability for health systems. However, a synthesis of the economic evidence on Y-90 TARE and ImCRC is lacking. In this sense, a systematic review is the most accurate methodology to identify the available information on a topic since it provides a synthesis of the results through a critical process of organized search. Also, previously published reviews of economic evaluations in CRC did not include Y-90 TARE [15] or included first-line systemic treatments [16]. Moreover, given the positive clinical evidence [10, 17] of Y-90 TARE therapy in reducing tumour burden in ImCRC patients, it is critical to explore and summarize the evidence on the economic benefits of Y-90 TARE therapy in these population. Given that a systematic review of the economic evaluations provides a synthesis of the available economic studies on health interventions to facilitate evidence-based decision-making, we sought to conduct a review of the evidence. Thus, the aim of this study was to identify, and review published economic evaluations of Y-90 TARE for the treatment of lmCRC.

## Methods

### Search strategy and identification of studies

A systematic review of economic evaluations of Y-90 TARE in lmCRC published in the literature up to May 2021 was conducted. The study followed the Preferred Reporting Items for Systematic Reviews and Meta-Analyses (PRISMA) methodology [18, 19]. The search strategy was designed with the Population, Intervention, Comparison, Outcomes (PICO) methodology using Boolean terms relating to lmCRC (Appendix 1). This systematic review was not registered on PROSPERO database.

The searched included databases (Medline through PubMed, Embase, The Cochrane Library and MEDES), health technology assessment agencies (European Network for Health Technology Assessment [EUnetHTA], Network of Health Technology Assessment Agencies [REDETS], and the National Institute for Health and Care Excellence [NICE]), and communications to international conferences (Cardiovascular and Interventional Radiological Society of Europe [CIRSE]; European Conference on Interventional Oncology [ECIO], European Association of Nuclear Medicine [EANM], Society of Interventional Oncology [SIO], International Society for Pharmacoeconomics and Outcomes Research [ISPOR], European Congress of Radiology [ECR], and Society of Nuclear Medicine and Molecular Imaging [SNMMI]). There was no limitation by type of economic evaluation study or year of publication, except for communications presented at congresses, for which a limitation to a 5-year period was applied.

### Eligibility criteria and article screening

The inclusion criteria considered only studies that performed an economic evaluation of Y-90 TARE, either as a single treatment, combination, or part of a treatment sequence, regardless of the treatment line, disease, or comparator. Studies that did not comply with the inclusion criteria were excluded. The eligibility criteria were applied first to the titles and abstracts of publications and then to the full texts of selected studies. Two authors (NE and IO) independently screened and selected studies for inclusion against the eligibility criteria. Any discrepancies after the review were resolved through discussion and a consensus meeting.

### Data extraction and data synthesis

Data was extracted by two authors (NE and IO) using a pre-specified data collection template which included these parameters: author(s), year and country of publication, patient characteristics, assessed comparative alternatives, types of Y-90 microspheres, type of economic evaluation, perspective, time horizon, type of model, cost estimation, health outcomes, and cost-effectiveness results. The type of economic evaluation was distinguished as either full (e.g., cost-effectiveness-analysis [CEA] and cost-utility analysis [CUA]) or partial (e.g., cost-analysis [CA]) economic evaluation. Cost estimates were extracted as reported in the publication (original cost) and then converted to international dollars ($US PPP) to eliminate the differences in the purchasing power between the different currencies of the countries on the selected publications. For this purpose, the original reported costs were updated to 2020 by applying the annual consumer price inflation (corresponding to the country of the publication) published by the World Bank [20]. And then, the purchasing power parity factor (PPP) was applied to transformed the respective costs to $US PPP ($US PPP, 2020) [21].

### Quality assessment

The quality of economic evaluations was assessed according to the Consolidated Health Economic Evaluation Reporting Standards (CHEERS) checklist 2022 version [22], which includes a 28-item checklist. The score assigned were 1 if the explicit parameters were described in the studies, or a score of 0 if they were not. The full and the partial economic evaluations were evaluated based on the 28-item checklist. An internal classification criterion was established to assess and categorise the included studies into low (< 50%), medium (50–80%), and high (> 80%) quality, according to the items fulfilled.

## Results

### Study selection and overall characteristics

**Fig. 1:**
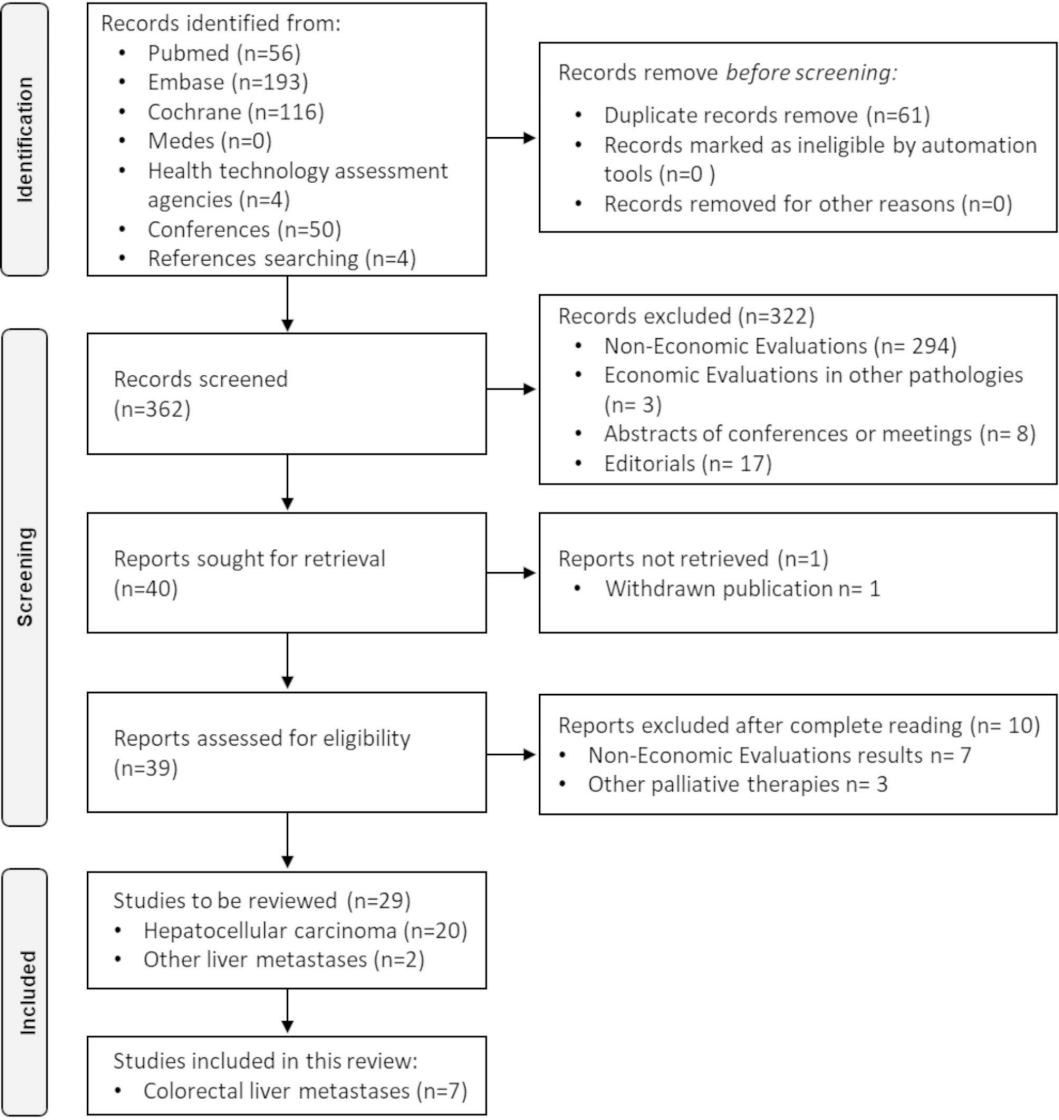
Bibliographic selection based on the PRISMA criteria

Overall, 423 potential studies were identified for titles and abstracts screening. After de-duplication and compliance with the inclusion criteria, 29 studies were selected for full-text review. Of these, 22 studies were excluded as they focused on hepatocellular carcinoma (n = 20), metastasis of neuroendocrine tumours of hepatic origin (n = 1), and intrahepatic cholangiocarcinoma (n = 1). This resulted in the selection of seven publications on lmCRC. The PRISMA diagram is illustrated in Fig. 1. Among the seven included studies, five (71%) were full economic evaluations [23–27] and two (29%) studies were partial evaluations [28, 29]. According to the CHEERS checklist, four articles had a medium–high quality assessment (mean scores of 88%) and three communications in congress were of lower quality assessment (mean score of 60%) because of their lesser breadth of data. An overview of the included studies (n = 7) is provided in Table 1.

**Table 1:** Quality assessment using the CHEERS 2022 statement checklist

	Section/Item	FULL ECONOMIC EVALUATIONS					PARTIAL ECONOMIC EVALUATIONS	
		Italy	United Kingdom				USA	United Kingdom
		Cosimelli, 2013 [24] ^a^	Bester, 2013 [23] ^a^	Pennington, 2015 [25] ^b^	Brennan, 2020 [26] ^b^	Loveman, 2014 [27] ^b^	Fusco, 2017 [28] ^a^	Dhir, 2018 [29] ^b^
1	Title	1	1	1	1	1	1	1
2	Abstract	0	0	1	1	1	0	1
3	Background and objectives	1	1	1	1	1	1	1
4	Health economics analysis plan	0	0	0	0	0	0	0
5	Study population	1	1	1	1	1	1	1
6	Setting and location	1	1	1	1	1	1	1
7	Comparators	1	1	1	1	1	1	1
8	Perspective	1	1	1	1	1	1	1
9	Time horizon	0	0	1	1	1	1	1
10	Discount rate	0	1	1	1	1	0	0
11	Selections of outcomes	1	1	1	1	1	1	1
12	Measurement of outcomes	0	0	1	1	1	0	1
13	Valuation of outcomes	1	1	1	1	1	1	1
14	Measurement and valuation of resources and cost	0	0	1	1	1	0	1
15	Currency, price date, and conversion	0	1	0	1	1	1	0
16	Rationale and description of model	0	0	1	1	1	0	1
17	Analytic methods and assumptions	0	0	1	1	1	1	1
18	Characterizing heterogeneity	0	1	1	1	1	0	0
19	Characterizing distributional effects	1	1	1	1	1	1	1
20	Characterizing uncertainty	0	1	1	1	1	0	0
21	Approach to engagement with patients and others affected by the study	0	0	0	1	1	0	1
22	Study parameters	0	1	1	1	1	0	1
23	Summary of main results	1	1	1	1	1	1	1
24	Effect to uncertainly	0	1	1	1	1	0	1
25	Effect of engagement with patients and others affected by the study	0	0	0	0	0	0	0
26	Study findings, limitations, generalizability, and current knowledge	0	1	1	1	1	0	1
27	Source of funding	0	1	1	1	1	0	0
28	Conflicts of interest	0	0	1	1	1	0	1
	Total	10	18	24	26	26	13	21
	% (n)	36%	64%	86%	93%	93%	52%	84%

^a^Oral communications or abstracts. b. Article

### Full economic evaluations characteristics

All the included studies categorized as full economic evaluations (n = 5) [23–27] were published from a European perspective. The study population were patients with unresectable lmCRC, with mainly hepatic predominance, and refractory or intolerant to chemotherapy. All the included studies focused on Y-90 resin microspheres. Four studies [23–26] compared Y-90 TARE monotherapy to best supportive care (BSC). The fifth study [27] compared the combination of Y-90 TARE with hepatic artery infusion with floxuridine (HAI) versus HAI. All five studies performed a CUA analyses. Four studies [23–26] used Markov modelling, and one study [27] used a survival-based model. Three of the five studies reported a lifetime horizon [25–27] while two studies did not report a time horizon [23, 24]. Four (4/5, 80%) [23–26] of the studies evaluated a payer’s perspective and the fifth study (1/5, 20%) [27] focused on the social perspective. The outcome measures included costs, life year gained (LYG), quality-adjusted life years (QALY), incremental cost-effectiveness ratio (ICER), incremental cost-utility ratio (ICUR), and willingness-to-pay (WTP). The characteristics of the full economics evaluations are summarized in Table 2.

**Table 2:** Descriptive analysis of full economic evaluations for liver metastases from colorectal cancer (lmCRC)

Author, year, publication type and country	Patient’s characteristics	Treatments		Analysis type/Model	Perspective/Time horizon	Cost	Outcomes
		**Comparators**	**Microspheres**				
Y-90 TARE vs. BSC							
Bester, 2013 [23] *Communication at congress* United Kingdom	lmCRC unresectable hepatic-predominant, refractory to chemotherapy ^a^	Y-90 TARE vs. BSC	Y-90 resin microspheres	CUA / Markov	Payer / ND	Direct cost (medical): Y-90 TARE (acquisition, preparation, and procedure), BSC treatment, monitoring, AE management and palliative care.	Cost, LYG, QALY, ICER (€/LYG), ICUR (€/QALY) and WTP (£30.000/QALY)
Cosimelli, 2013 [24] *Communication at congress* Italy	lmCRC unresectable hepatic-predominant, refractory to chemotherapy ^a^	Y-90 TARE vs. BSC	Y-90 resin microspheres	CUA / Markov	Payer / ND	Direct cost (medical): Y-90 TARE (acquisition, preparation, and procedure), additional chemotherapy, AE management and palliative care.	Cost, LYG, QALY, ICER (€/LYG), ICUR (€/QALY) and WTP (€50.000/QALY)
Pennington, 2015 [25] *Original article* United Kingdom	lmCRC unresectable hepatic-predominant, refractory to chemotherapy ^b^	Y-90 TARE vs. BSC	Y-90 resin microspheres	CUA / Markov	Payer / lifetime	Direct cost (medical): Y-90 TARE (preparation and procedure), BSC treatment, monitoring, additional treatment, AE management and palliative care.	Cost, LYG, QALY, ICER (€/LYG), ICUR (€/QALY) and WTP (£30.000/QALY)
Brennan, 2020 [26] *Original article* United Kingdom	lmCRC unresectable hepatic-predominant, refractory/intolerant to chemotherapy ^b^	Y-90 TARE vs. BSC	Y-90 resin microspheres	CUA / Markov	Payer/ Lifetime	Direct cost (medical): Y-90 TARE (preparation and procedure), treatment, AE management and palliative care costs.	Cost, LYG, QALY, ICER (€/LYG), ICUR (€/QALY) and WTP (£30.000/QALY)
Y-90 TARE vs. HAI							
Loveman, 2014 [27] *Systematic review an economic evaluation* United Kingdom	lmCRC surgically unresectable ^c^	Y-90 TARE + HAI vs. HAI	Y-90 resin microspheres	CUA / Partitioned survival model	Payer and social/ Lifetime	Direct cost (medical): Treatment, post- treatment, monitoring, and palliative care.	Cost, LYG, QALY, ICER (€/LYG), ICUR (€/QALY) and WTP (£30.000/QALY)

AE: adverse event, BSC: best supportive care, CUA: cost-utility analysis, HAI: hepatic artery infusion with floxuridine, ICER: incremental cost-effectiveness ratio; ICUR: incremental cost-utility ratio; QALY: quality-adjusted life years, lmCRC: liver metastases from colorectal cancer, LYG: life year gained; ND: no data, Y-90 TARE: transarterial radioembolization with yttrium 90; WTP: willingness-to-pay

a. The sample size was not included in the analysis. b. The sample size reported in the analysis considered the retrospective data from *Bester et al. 2012* [30] (Y-90 TARE n = 224; BSC = 51). c. The sample size reported in the analysis considered the clinical trial data from *Grey et al. 2001* [32] (Y-90 TARE + HAI n = 36; HAI n = 34)

### Y-90 TARE versus BSC

BSC therapy was one of the comparators evaluated in four [23–26] out of the five studies. Only one study, *Pennington et al. 2015* [25] defined BSC therapy, as a treatment that included chemotherapy, biological agents, and/or other interventional procedures other than Y-90 TARE. The study by *Brennan et al. 2020* [26], described BSC as a therapy providing 4 to 6 month survival. The last two publications [23, 24] did not define BSC treatment and corresponded to communications in congress.

The Markov modelling simulated three states of transition disease (pre-progression, post-progression, and death) in the four studies [23–26]. The overall survival (OS) was based on *Bester et al. 2012* [30], a retrospective study of Y-90 TARE versus BSC in patients refractory to chemotherapy. Given *Bester et al. 2012* [30], did not report progression-free survival (PFS), the assumptions of transition disease were informed. The utilities used were based on *Hoyle et al. 2013* [31], an economic evaluation conducted by NICE on the treatments of lmCRC after a first line chemotherapy. The costs reported were similar in the four studies [23–26] and included direct medical costs.

### Y-90 TARE associated with HAI versus HAI

A corresponding study by *Loveman et al. 2014* [27] reported an economic evaluation comparing Y-90 TARE plus HAI (Y-90 TARE + HAI) versus HAI. HAI was defined as the infusion of floxuridine during 12 days with repetition at monthly intervals [27]. The source of efficacy, OS and PFS, was based on the clinical trial by *Grey et al. 2001* [32], which evaluated Y-90 TARE + HAI versus HAI in patients with unresectable bilobular liver metastases from primary large bowel adenocarcinoma. The utilities applied were based on studies by *Wiering et al. 2010* [33], *Krabbe et al. 2004* [34], and *Tappenden et al. 2014* [35].

### Full economic evaluations results

The costs and health outcomes reported in the five studies were homogeneous (Table 3). The results of the complete economic evaluations were analysed according to the comparators.

**Table 3:** Results of publications of full economics evaluations for liver metastases from colorectal cancer (lmCRC)

Author, year publication (year cost)	Comparators	Costs		Health Outcomes		Ratio cost / Outcome’s health			
		**Original cost**	**Adjusted to $US PPP** [21]	**LYG**	**QALY**	**ICER**	**ICUR**	**ICER** **$US PPP/LYG**	**ICUR** **$US PPP /QALY**
						**WTP**			
**Y-90 TARE vs. BSC**									
**Bester, 2013** [23] (2012)	Y-90 TARE	£ 35,487	39,221	2.09	1.50	Y-90 TARE vs. BSC	Y-90 TARE vs. BSC	Y-90 TARE vs. BSC	Y-90 TARE vs. BSC
	BSC	£ 12,730	14,069	0.97	0.69	£ 20,323	£ 28,216	22,461	31,185
	Difference Δ	Δ £ 22,757	Δ 25,151	Δ 1.12	Δ 0.81	WTP (£30,000/QALY): 57%			
**Cosimelli, 2013** [24] (2012)^a^	Y-90 TARE	€ 39,973	41,099	2.12	1.52	Y-90 TARE vs. BSC	Y-90 TARE vs. BSC	Y-90 TARE vs. BSC	Y-90 TARE vs. BSC
	BSC	€ 15,347	15,779	0.98	0.70	ND	€ 29,850	ND	30,691
	Difference Δ	Δ € 24,626	Δ 25,320	Δ 1.35	Δ 0.83	WTP (€50,000/QALY): 57%			
**Pennington, 2015** [25] (2013)^a^	Y-90 TARE	£ 35,487	38,592	2.09	1.50	Y-90 TARE vs. BSC	Y-90 TARE vs. BSC	Y-90 TARE vs. BSC	Y-90 TARE vs. BSC
	BSC	£ 12,730	13,844	0.97	0.69	£ 20,323	£ 28,216	22,101	30,684
	Difference Δ	Δ £ 22,757	Δ 24,748	Δ 1.12	Δ 0.81	WTP (£30,000/QALY): 57%			
**Brennan, 2020** [26] (2019)	Y-90 TARE	£ 34,168	34,810	ND	1.50	Y-90 TARE vs. BSC	Y-90 TARE vs. BSC	Y-90 TARE vs. BSC	Y-90 TARE vs. BSC
	BSC	£ 15,268	15,268	ND	0.69	£ 18,900	£ 23,435	19,255	23,875
	Difference Δ	Δ £ 18,900	Δ 19,255	Δ ND	Δ 0.81	WTP (£30,000/QALY): 56%			
**Y-90 TARE vs. HAI**									
**Loveman, 2014** [27] (2012)	Y-90 TARE + HAI	£ 18,955	20,949	1.86	1.41	Y-90 TARE + HAI vs. HAI	Y-90 TARE + HAI vs. HAI	Y-90 TARE + HAI vs. HAI	Y-90 TARE + HAI vs. HAI
	HAI	£ 6,010	6,642	1.49	1.06	£ 35,225	£ 37,303	38,931	41,228
	Difference Δ	Δ £ 12,945	Δ 14,307	Δ 0.37	Δ 0.35	WTP (£30,000/QALY): 26%			

BSC: best supportive care (include chemotherapy, biological agents, and further interventional procedures), HAI: hepatic artery infusion with floxuridine, ICER: cost-effectiveness incremental ratio, ICUR: incremental cost-utility ratio, lmCRC: liver metastases from colorectal cancer, LYG: life years gained, ND: no data, OS: overall survival, QALY: quality-adjusted life years, Y-90 TARE: transarterial radioembolization with yttrium 90, WTP: willingness-to-pay, $US PPP: Purchasing-power-parity exchange rates for the year 2020

a. In case of unspecified cost year, an estimation of proposed cost reference source was used: years 2012 and 2013 were adopted for *Cosimelli et al.*. and *Pennington et al.*. respectively

### Y-90 TARE versus BSC

The four [23–26] studies reported higher costs with Y-90 TARE therapy than BSC, and the incremental costs ranged between 19,255 [26] and 25,320 [24] $US PPP [23–26]. The health outcomes reported for patients showed a benefit of Y-90 TARE over BSC in terms of LYG and QALY in the four studies. LYG range between 2.09 and 2.12, and QALY range between 1.50 and 1.52. The ICERs of Y-90 TARE versus BSC oscillated between £ 18,900/LYG (£, 2019) (19,255 $US PPP/LYG) to £ 20,323/LYG (£, 2012) (22,461 $US PPP/LYG) and ICURs between £ 23,435/QALY (£, 2019) (23,875 $US PPP/QALY) to £ 22,461/QALY (£, 2012) (31,185 $US PPP/QALY). The probability of Y-90 TARE being efficient was 56% [26] or 57% [23, 25] considering a cost-effectiveness threshold of £ 30,000/QALY, and 97% [24] considering a threshold of € 50,000/QALY.

### Y-90 TARE associated with HAI versus HAI

The study by *Loveman et al. 2014* [27] reported higher costs with Y-90 TARE + HAI therapy than with HAI alone (incremental cost of 14,307 $US PPP). The health outcomes reported were favourable for patients with Y-90 TARE + HAI, showing increases of 0.37 LYG and 0.35 QALY over HAI therapy alone. The study reported an ICER of £ 35,225 (£, 2012) (38,931 $US PPP/LYG), and an ICUR of £ 37,303 (£, 2012) (41,228 $US PPP/QALY). The probability of being efficient was 26% considering a cost-effectiveness threshold of £ 30,000/QALY.

### Assessment of quality of full economic evaluations

The quality of the included studies, classified as full economic evaluations, was assessed as follows: three of the five studies (60%) [25–27] had a high score, with a mean compliance of 90% of the 28 evaluated items. One of the five studies (20%) had a moderate score (mean compliance of 64%) [23]. The last publication (20%) had a mean compliance of 36% [24].

### Partial economic evaluations characteristics

Two publications [28, 29] included a congress communication [28] and an original article [29] were categorized as partial economic evaluations. Each study was from two perspectives: European [28] and the United States [29]. The population characteristics in the study by *Fusco et al. 2017* [28] corresponded to a first line of treatment, based on the FOXFIRE study [36] (patients with CRC metastases, without prior chemotherapy treatment, unsuitable for resection or ablation). The population characteristics on *Dhir et al. 2018* [29] study corresponded to a second line of treatment, in patients with isolated, unresectable lmCRC, refractory to chemotherapy. Regarding the evaluated microspheres, only one (*Fusco et al. 2017* [28]) of the two studies, referred to Y-90 resin microspheres, the other study (*Dhir et al. 2018* [29]) did not specify the type of microspheres. The comparator treatments were FOLFOX (defined by the association of oxaliplatin, 5-fluorouracil and folinic acid) [28] and the HAI with floxuridine associated with recent chemotherapy (MDR, defined by multi-drug regimens including oxaliplatin and/or irinotecan ± biological treatments) [29]. Regarding the pharmacoeconomic parameters, both studies were CAs, and the time horizon reported were two years [29] and three years [28]. The stages of the study population, the comparators, and the outcome measures considered in the partial economic evaluations are summarized in Table 4.

**Table 4:** Descriptive analysis of partial economic evaluations for liver metastases from colorectal cancer (lmCRC)

Author, year, publication type and country	Patient’s characteristic	Treatments		Analysis type/Clinical source	Perspective/Time horizon	Outcomes
		**Comparators**	**Microspheres**			
Fusco, 2017 [28] *Communication at congress* United Kingdom	lmCRC not suitable for resection/ablation; chemotherapy-naïve (First line treatment) ^a^	Y-90 TARE + FOLFOX vs. FOLFOX	Y-90 resin microspheres	CA / FOXFIRE clinical trial	Payer/ 3 years	Direct cost (medical) on Primary care resource. QoL
Dhir, 2018 [29] *Original article* USA	lmCRC liver-only unresectable (Second line treatment) ^b^	Y-90 TARE + MDR vs. HAI + MDR	ND	CA / Retrospective study	Payer/ 2 years	Direct cost (medical) estimated retrospectively by the consumption of hospital resources. OS

HAI: hepatic artery infusion (pump) with floxuridine, FOLFOX: folinic acid, fluorouracil and oxaliplatin, lmCRC: liver metastases from colorectal cancer, OS: overall survival, QoL: quality of life, MDR: multi-drug regimens including oxaliplatin and/or irinotecan ± biological treatments, Y-90 TARE: transarterial radioembolization with yttrium 90

a. Chemotherapy-naïve metastatic colorectal cancer patients with liver metastases. The sample size reported in this analysis considered the clinical trial data from FOXFIRE study [36] (N = 364), and then specified patients treated according to *Wasan et al. 2017* [40] (Y-90 TARE + FOLFOX n = 167; FOLFOX n = 169). b. Pre-treated patients with a heavy liver tumour burden (median of 10 lesions and almost 40% of liver parenchymal replacement by tumour). The sample size reported in this analysis considered the clinical data from *Dhir et al. 2018* [29] study (Y-90 TARE n = 49; HAI n = 48)

### Partial economic evaluations results

In the first line of treatment, the CA by *Fusco et al. 2017* [28] reported higher treatment costs with Y-90 TARE plus FOLFOX than FOLFOX, although Y-90 TARE did not significantly increase primary care resource consumption. The incremental cost in the first year was £ 51.79 (£, 2017) (54.85 $US PPP) and was £ 56.38 (£, 2017) (59.72 $US PPP) cumulatively over three years. In the second line of treatment, the CA by *Dhir et al. 2018* (Y-90 TARE + MDR vs. HAI + MDR) [29] reported an average higher cost with Y-90 TARE + MDR ($ 39,092 [41,238 $US PPP]) than HAI + MDR ($ 29,479 [31,097 $US PPP]), although the study did not demonstrate statistically significant differences (p = 0.296) on these results (Table 5).

**Table 5:** Results of publications of partial evaluations for liver metastases from colorectal cancer (lmCRC)

Author, year publication (year cost)	Stage	Comparators	Costs		Health outcomes
			Original cost	Adjusted to $US PPP	
Fusco, 2017 [28] (2016)	lmCRC (First line treatment)^a^	Y-90 TARE + FOLFOX	£ 209.44^b^	221.83 $US PPP	Δ QoL utilities (EQ-5D-3 L): -0.001 at 2 months (CI 95%: -0.05, 0.05), -0.03 at 12 months (-0.16, 0.09), 0.03 at 24 months, (-0.09, 0.16), and -0.03 at 36 months (-0.20, 0.14).
		FOLFOX	£ 158.85^b^	168.25 $US PPP	
			Δ Cost at first year: £ 51.79^c^ Δ Cost by 3 years: £ 56,38^c^	54,85 $US PPP 59,72 $US PPP	
Dhir, 2018 [29] (2018)^c^	lmCRC (Second line treatment)^d^	Y-90 TARE + MDR	$ 39,092 (n = 13; 2 days)	41,238 $US PPP	Median OS (since lmCRC diagnosis) Y-90 TARE: 16.3 months (12.2–22.4) HAI: 31.2 months (20.8–35.5)
		HAI + MDR	$ 29,479 (n = 21; 9 days)	31,097 $US PPP	
			Δ Cost (median): $15,948	16,824 $US PPP	

HAI: hepatic artery infusion with floxuridine, FOLFOX: folinic acid, fluorouracil and oxaliplatin, lmCRC: liver metastases from colorectal cancer, OS: overall survival, QoL: quality of life, MDR: multi-drug regimens including oxaliplatin and/or irinotecan ± biological treatments, Y-90 TARE: transarterial radioembolization with yttrium 90, $US PPP: Purchasing-power-parity exchange rates for the year 2020

a. Chemotherapy-naïve metastatic colorectal cancer patients with liver metastases. b. Only Primary care resource was considered. c. Cost year not specified, estimated from the proposed cost reference sources. d. Pre-treated patients with a heavy liver tumour burden (median of 10 lesions and almost 40% of liver parenchymal replacement by tumour)

### Assessment of quality of partial economic evaluations

One of the two publications [28] presented a medium score, with an average compliance of 52% with the 28 items evaluated. The other publication [29] (50%) presented a high score, with an average compliance of 84% with the 28 items evaluated.

## Discussion

This study is part of a systematic review on the economic evaluations of Y-90 TARE therapy in liver neoplasms [37]. This research focuses on the evidence of economic evaluations, both full and partial, of Y-90 TARE therapy in the treatment of patients with lmCRC. This review identified 7 economic evaluations (5 full and 2 partial) that assessed the Y-90 TARE therapy as an intervention for treating lmCRC.

The inclusion of Y-90 TARE therapy was associated with additional costs [23–29], mainly because it was compared to well-established, low-priced chemotherapy drugs such as HAI [27, 29] and FOLFOX [28]) or to BSC [23–26], a symptom management therapy. Despite the additional cost (range 16,824 [29] to 25,320 [24] $US PPP), Y-90 TARE therapy has demonstrated advantages in improving hospital efficiencies such as reducing hospital stay (2 days for Y-90 TARE vs. 9 days for HAI) [29]; improving health outcomes (Y-90 TARE versus BSC [23–26] or HAI [27]), improving LYG (Y-90 TARE versus BSC: 1.12 [23, 25] to 1.35 [24], and Y-90 TARE versus HAI 0.37), and improving QALYs (Y-90 TARE versus BSC: 0.81 [23, 25, 26] to 0.83 [24] and Y-90 TARE + HAI versus HAI: 0.35 [27]). Although, the retrospective study of Y-90 TARE vs. HAI [29] showed a higher OS for HAI (16.3 vs. 31.2 months), the study reported a lower probability of survival as more patients in the Y-90 TARE group had a prior liver resection at the time of diagnosis.

Likewise, Y-90 TARE therapy could be considered a cost-effective option over BSC, for treating patients with lmCRC (chemotherapy-refractory and hepatic predominance), with costs lower than 31,185 $US PPP/QALY (22,461 $US PPP/LYG) [23] in at least 57% of cases (with a WTP at threshold of £30,000/QALY) when considering the payer perspective. However, the cost-effectiveness range increased to 41,228 $US PPP/QALY (38,931 $US PPP/LYG) [27], while decreasing the probability of WTP up to 26%, when considering the social perspective and HAI.

To provide more context to the economic evaluation outcomes, we also reviewed the clinical evidence. The CA by *Fusco et al. 2017* [28] evaluated the use of Y-90 TARE in first-line treatment for chemotherapy-naïve patients, and identified limited information on the primary care resources costs as a limitation. The remaining economic studies [23–27, 29] evaluated the use of Y-90 TARE in successive lines of treatment for chemotherapy refractory patients, drawing clinical data from two retrospective studies [29, 30] and one clinical trial [32]. The first retrospective study by *Bester et al. 2012* [30], had a representative population (N = 339) for Y-90 TARE and was used in four [23–26] full economic evaluations. The second retrospective study *Dhir et al. 2018* [29] was used to calculate treatment cost of HAI and Y-90 TARE in the same reference [29] and evaluated a smaller population (N = 49). Furthermore, the clinical trial by *Grey et al. 2001* [32]evaluated a smaller population (N = 35) and was used to compare Y-90 TARE + HAI versus HAI [27].

Given the indications for treatment with Y-90 TARE in the *Society of Interventional Radiology* [38], which focuses on including patients with hepatic and surgically unresectable liver neoplasms, the choice of the patient is relevant for an optimal outcome. As such, the combination of Y-90 TARE with a systemic chemotherapy treatment as the first-line of the treatment of patients with unresectable lmCRC is not recommended [39]. However, the addition of Y-90 TARE to standard second-line chemotherapy (as demonstrated in the phase III EPOCH clinical trial) [10] has shown improved PFS and hepatic PFS, further supporting the advantage on the cost-effectiveness of Y-90 TARE therapy in patients with unresectable lmCRC.

This review has several limitations. First, there is no standardized definition of BSC therapy. Thus, this lack of definition coupled with the fact that three publications [24, 25, 29] did not specify the reference dates for costs, potentially contributing to the variability of the results, including direct health costs (19,255 to 25,320 $US PPP). Second, the studies reported costs in different currencies and reference years, limiting the comparability of results, which were converted to 2020 ($US PPP costs) to address this issue. Lastly, while this is a global systematic review, most economic evaluations were conducted from the European perspective, which may limit the external validity of our review to other countries. As such, the authors recommend using a system to ensure the transferability of economic evaluations before applying the results extracted from them.

## Conclusion

This systematic review examines economic evaluations of Y-90 TARE for the treatment of lmCRC and highlights that Y-90 TARE could be a cost-effective therapy, either as a monotherapy or in combination with a systemic therapy, for the treatment of patients with lmCRC. The evaluation of health technologies such as Y-90 TARE provides a tool to aid in decision-making to maximize health benefits for lmCRC patients and in resource allocation for health systems. However, given the limited number of global economic evaluations on Y-90 TARE in treating ImCRC (n = 7), further research is recommended on the economic evaluations on Y-90 TARE vs. alternative therapies in treating ImCRC from the societal perspective.

## Electronic supplementary material

Below is the link to the electronic supplementary material.

Supplementary Material 1

## Data Availability

The datasets used, generated, and/or analysed during the current study are not publicly available due to commercial restrictions but are available from the corresponding author on reasonable request.

## Abbreviations

Y-90 TARE: Transarterial radioembolization with yttrium-90
AE: Adverse Events
ASCO: American Society of Clinical Oncology
BSC: Best supportive care
CA: Cost-analysis
CHEERS: Consolidated Health Economic Evaluation Reporting Standards
CIRSE: Cardiovascular and Interventional Radiological Society of Europe
CRC: Colorectal cancer
CUA: Cost-utility-analysis
EANM: European Association of Nuclear Medicine
ECIO: European Conference on Interventional Oncology
ECR: European Congress of Radiology
ESMO: European Society for Medical Oncology
ESMO: European Society of Medical Oncology
EUNetHTA: European Network for Health Technology Assessment
FOLFOX: Folinic acid, fluorouracil and oxaliplatin
HAI: Hepatic artery infusion
ICER: Incremental cost-effectiveness ratio
ICUR: Incremental cost-utility ratio
ISPOR: International Society for Pharmacoeconomics and Outcomes Research
lmCRC: Liver metastases from colorectal cancer
LYG: Life-years gained
MDR: Multi-drug regimens including oxaliplatin and/or irinotecan ± biological treatments
NCCN: National Comprehensive Cancer Network
NICE: National Institute for Health and Clinical Excellence
OS: Overall survival
PFS: Progression-free survival
PICO: Population, Intervention, Comparison, Outcomes
PPP: Purchasing power parity
PRISMA: Preferred Reporting Items for Systematic Reviews and Meta-Analyses
QoL: Quality of life
QALY: Quality-adjusted life year
REDETS: Network of Health Technology Assessment Agencies
SIO: Society of Interventional Oncology
SNMMI: Society of Nuclear Medicine and Molecular Imaging
WTP: Willingness-to-pay
