# Supplementary material for: Economic evaluations of radioembolization with yttrium-90 microspheres in liver metastases of colorectal cancer: a systematic review
Source: BMC Gastroenterol. 2023 May 24;23:181. doi: 10.1186/s12876-023-02793-5 (PMC10210491; doi:10.1186/s12876-023-02793-5)
Supplement: Supplementary file 1 — Supplementary Material 1 [file 12876_2023_2793_MOESM1_ESM.docx]

**SUPPLEMENTARY INFORMATION**

BMC GASTROENTEROLOGY

ECONOMIC EVALUATIONS OF RADIOEMBOLIZATION WITH YTTRIUM-90 MICROSPHERES IN LIVER METASTASES OF COLORECTAL CANCER: A SYSTEMATIC REVIEW

Alonso JC^1^, Casans I^2^, González FM^3^, Fuster D^4^, Rodríguez A^5^, Sánchez N^4^, Oyagüez I^6^, Williams AO^7^, Espinoza N^6^

^1^Nuclear Medicine Department, Hospital Gregorio Marañón, Madrid, Spain

^2^Nuclear Medicine Department, Hospital Clínico Universitario, Valencia, Spain

^3^Nuclear Medicine Department, Hospital Universitario Central, Asturias, Spain

^4^Nuclear Medicine Department, Hospital Clinic, Barcelona, Spain

^5^Nuclear Medicine Department, Hospital Virgen de las Nieves, Granada, Spain

^6^Pharmacoeconomics & Outcomes Research Iberia (PORIB), Madrid, Spain

^7^Boston Scientific Marlborough, Massachusetts, USA

**Appendix 1. Terminology of searching strategy in PubMed**

| **Search** | | | **Terms** | **Search strategy** | **Results** |
| --- | --- | --- | --- | --- | --- |
| #1 | Hepatocarcinoma | | | "carcinoma hepatocellular"[MeSH Terms] OR ("carcinoma"[All Fields] AND "hepatocellular"[All Fields]) OR "hepatocellular carcinoma"[All Fields] OR "hepatocarcinoma"[All Fields] OR "hepatocarcinomas"[All Fields] | 127,003 |
| #2 | Hepatic neoplasms | | | "liver neoplasms"[MeSH Terms] OR ("liver"[All Fields] AND "neoplasms"[All Fields]) OR "liver neoplasms"[All Fields] OR ("hepatic"[All Fields] AND "neoplasms"[All Fields]) OR "hepatic neoplasms"[All Fields] | 238,813 |
| #3 | Primary liver tumour | | | ("primaries"[All Fields] OR "primary"[All Fields]) AND ("liver neoplasms"[MeSH Terms] OR ("liver"[All Fields] AND "neoplasms"[All Fields]) OR "liver neoplasms"[All Fields] OR ("liver"[All Fields] AND "tumour"[All Fields]) OR "liver tumour"[All Fields]) | 35,870 |
| #4 | Primary liver tumors | | | ("primaries"[All Fields] OR "primary"[All Fields]) AND ("liver neoplasms"[MeSH Terms] OR ("liver"[All Fields] AND "neoplasms"[All Fields]) OR "liver neoplasms"[All Fields] OR ("liver"[All Fields] AND "tumors"[All Fields]) OR "liver tumors"[All Fields]) | 37,233 |
| #5 | Liver metastases | | | ("liver"[MeSH Terms] OR "liver"[All Fields] OR "livers"[All Fields] OR "liver s"[All Fields]) AND ("metastasation"[All Fields] OR "metastasic"[All Fields] OR "metastasing"[All Fields] OR "metastasise"[All Fields] OR "metastasised"[All Fields] OR "metastasises"[All Fields] OR "metastasising"[All Fields] OR "metastasization"[All Fields] OR "metastasizes"[All Fields] OR "metastasizing"[All Fields] OR "neoplasm metastasis"[MeSH Terms] OR ("neoplasm"[All Fields] AND "metastasis"[All Fields]) OR "neoplasm metastasis"[All Fields] OR "metastase"[All Fields] OR "metastases"[All Fields] OR "metastasize"[All Fields] OR "metastasized"[All Fields]) | 55,403 |
| #6 | Secondary liver cancer | | | ("neoplasm metastasis"[MeSH Terms] OR ("neoplasm"[All Fields] AND "metastasis"[All Fields]) OR "neoplasm metastasis"[All Fields] OR "secondaries"[All Fields] OR "secondary"[MeSH Subheading] OR "secondary"[All Fields]) AND ("liver neoplasms"[MeSH Terms] OR ("liver"[All Fields] AND "neoplasms"[All Fields]) OR "liver neoplasms"[All Fields] OR ("liver"[All Fields] AND "cancer"[All Fields]) OR "liver cancer"[All Fields]) | 61,608 |
| #7 | #1OR#2OR#3  OR#4OR#5 | | | (((((hepatocarcinoma) OR (hepatic neoplasms)) OR (primary liver tumour)) OR (primary liver tumors)) OR (Liver metastases)) OR (secondary liver cancer) | 280,095 |
| #8 | Carcinoma Hepatocellular | | | "carcinoma hepatocellular"[MeSH Terms] OR ("carcinoma"[All Fields] AND "hepatocellular"[All Fields]) OR "hepatocellular carcinoma"[All Fields] OR ("carcinoma"[All Fields] AND "hepatocellular"[All Fields]) OR "carcinoma hepatocellular"[All Fields] | 125,059 |
| #9 | Carcinoma Hepatocelular | | | ("carcinoma"[MeSH Terms] OR "carcinoma"[All Fields] OR "carcinomas"[All Fields] OR "carcinoma s"[All Fields]) AND "Hepatocelular"[All Fields] | 202 |
| #10 | HCC | | | "HCC"[All Fields] | 61,653 |
| #11 | Intrahepatic cholangiocarcinoma | | | "cholangiocarcinoma"[MeSH Terms] OR "cholangiocarcinoma"[All Fields] OR ("intrahepatic"[All Fields] AND "cholangiocarcinoma"[All Fields]) OR "intrahepatic cholangiocarcinoma"[All Fields] | 16,164 |
| #12 | Colorectal metastasis | | | "Colorectal"[All Fields] AND ("metastasi"[All Fields] OR "neoplasm metastasis"[MeSH Terms] OR ("neoplasm"[All Fields] AND "metastasis"[All Fields]) OR "neoplasm metastasis"[All Fields] OR "metastasis"[All Fields]) | 27,139 |
| #13 | Colorectal metastases | | | "Colorectal"[All Fields] AND ("metastasation"[All Fields] OR "metastasic"[All Fields] OR "metastasing"[All Fields] OR "metastasise"[All Fields] OR "metastasised"[All Fields] OR "metastasises"[All Fields] OR "metastasising"[All Fields] OR "metastasization"[All Fields] OR "metastasizes"[All Fields] OR "metastasizing"[All Fields] OR "neoplasm metastasis"[MeSH Terms] OR ("neoplasm"[All Fields] AND "metastasis"[All Fields]) OR "neoplasm metastasis"[All Fields] OR "metastase"[All Fields] OR "metastases"[All Fields] OR "metastasize"[All Fields] OR "metastasized"[All Fields]) | 29,796 |
| #14 | Colorectal carcinoma | | | "colorectal neoplasms"[MeSH Terms] OR ("colorectal"[All Fields] AND "neoplasms"[All Fields]) OR "colorectal neoplasms"[All Fields] OR ("colorectal"[All Fields] AND "carcinoma"[All Fields]) OR "colorectal carcinoma"[All Fields] | 230,976 |
| #15 | Colorectal neoplasms | | | "colorectal neoplasms"[MeSH Terms] OR ("colorectal"[All Fields] AND "neoplasms"[All Fields]) OR "colorectal neoplasms"[All Fields] | 226,333 |
| #16 | Colon | | | "colon"[MeSH Terms] OR "colon"[All Fields] OR "colonic"[All Fields] OR "colons"[All Fields] OR "colon s"[All Fields] OR "colonal"[All Fields] OR "colonically"[All Fields] OR "colonitis"[All Fields] | 286,383 |
| #17 | Neuroendocrine tumours | | | "neuroendocrine tumours"[All Fields] OR "neuroendocrine tumors"[MeSH Terms] OR ("neuroendocrine"[All Fields] AND "tumors"[All Fields]) OR "neuroendocrine tumors"[All Fields] | 182,723 |
| #18 | #8OR#9OR#10OR#11OR#12OR#13OR#14OR#15OR#16OR#17 | | | (((((((((Carcinoma Hepatocellular) OR (Carcinoma Hepatocelular)) OR (HCC)) OR (intrahepatic cholangiocarcinoma)) OR (Colorectal metastasis)) OR (Colorectal metastases)) OR (Colorectal carcinoma)) OR (Colorectal neoplasms)) OR (colon)) OR (Neuroendocrine tumours) | 724,487 |
| #19 | #7OR#18 | | | **Population**  ((((((hepatocarcinoma) OR (hepatic neoplasms)) OR (primary liver tumour)) OR (primary liver tumors)) OR (Liver metastases)) OR (secondary liver cancer)) OR ((((((((((Carcinoma Hepatocellular) OR (Carcinoma Hepatocelular)) OR (HCC)) OR (intrahepatic cholangiocarcinoma)) OR (Colorectal metastasis)) OR (Colorectal metastases)) OR (Colorectal carcinoma)) OR (Colorectal neoplasms)) OR (colon)) OR (Neuroendocrine tumours)) | 836,222 |
| #20 | | Yttrium-90 | | "yttrium 90"[Supplementary Concept] OR "yttrium 90"[All Fields] OR "yttrium 90"[All Fields] | 2,219 |
| #21 | | 90Y | | "90Y"[All Fields] | 2,806 |
| #22 | | 90-Y | | "90-Y"[All Fields] | 2,174 |
| #23 | | Y90 | | "Y90"[All Fields] | 616 |
| #24 | | Y-90 | | "Y-90"[All Fields] | 496 |
| #25 | | 20OR#21OR  #22#23OR#24 | | ((((Yttrium-90) OR (90Y)) OR (90-Y)) OR (Y90)) OR (Y-90) | 5,166 |
| #26 | | radioembolization | | "radioembolic"[All Fields] OR "radioembolisation"[All Fields] OR "radioembolization"[All Fields] OR "radioembolizations"[All Fields] | 1,932 |
| #27 | | transarterial radioembolization | | ("transarterial"[All Fields] OR "transarterially"[All Fields]) AND ("radioembolic"[All Fields] OR "radioembolisation"[All Fields] OR "radioembolization"[All Fields] OR "radioembolizations"[All Fields]) | 589 |
| #28 | | transcatheter arterial radioembolization | | "transcatheter"[All Fields] AND ("arterialization"[All Fields] OR "arterializations"[All Fields] OR "arterialize"[All Fields] OR "arterialized"[All Fields] OR "arterializing"[All Fields] OR "arterially"[All Fields] OR "arterials"[All Fields] OR "arterie"[All Fields] OR "arteries"[MeSH Terms] OR "arteries"[All Fields] OR "arterial"[All Fields] OR "arteris"[All Fields] OR "artery"[All Fields] OR "arterious"[All Fields] OR "artery s"[All Fields] OR "arterys"[All Fields]) AND ("radioembolic"[All Fields] OR "radioembolisation"[All Fields] OR "radioembolization"[All Fields] OR "radioembolizations"[All Fields])v | 57 |
| #29 | | TARE | | "TARE"[All Fields] | 631 |
| #30 | | Selective internal radiation therapy | | ("select"[All Fields] OR "selectability"[All Fields] OR "selectable"[All Fields] OR "selected"[All Fields] OR "selecting"[All Fields] OR "selection s"[All Fields] OR "selection. genetic"[MeSH Terms] OR ("selection"[All Fields] AND "genetic"[All Fields]) OR "genetic selection"[All Fields] OR "selection"[All Fields] OR "selectional"[All Fields] OR "selections"[All Fields] OR "selective"[All Fields] OR "selectively"[All Fields] OR "selectives"[All Fields] OR "selectivities"[All Fields] OR "selectivity"[All Fields] OR "selects"[All Fields]) AND ("brachytherapy"[MeSH Terms] OR "brachytherapy"[All Fields] OR ("internal"[All Fields] AND "radiation"[All Fields] AND "therapy"[All Fields]) OR "internal radiation therapy"[All Fields]) | 4,261 |
| #31 | | SIRT | | "sirtuins"[MeSH Terms] OR "sirtuins"[All Fields] OR "sirt"[All Fields] | 11,911 |
| #32 | | #26OR#27OR  #28OR#29OR  #30OR#31 | | (((((radioembolization) OR (transarterial radioembolization)) OR (transcatheter arterial radioembolization)) OR (TARE)) OR (Selective internal radiation therapy)) OR (SIRT) | 17,859 |
| #33 | | sirtuins | | "sirtuins"[MeSH Terms] OR "sirtuins"[All Fields] OR "sirtuin"[All Fields] | 13,030 |
| #34 | | #32NOT#33 | | Radiembolization ((((((radioembolization) OR (transarterial radioembolization)) OR (transcatheter arterial radioembolization)) OR (TARE)) OR (Selective internal radiation therapy)) OR (SIRT)) NOT (sirtuins) | 6,798 |
| #35 | | #25OR#34 | | Radioembolization o Yttrium-90 (((((Yttrium-90) OR (90Y)) OR (90-Y)) OR (Y90)) OR (Y-90)) OR (((((((radioembolization) OR (transarterial radioembolization)) OR (transcatheter arterial radioembolization)) OR (TARE)) OR (Selective internal radiation therapy)) OR (SIRT)) NOT (sirtuins)) | 10,533 |
| #36 | | TheraSphere | | "therasphere"[All Fields] OR "theraspheres"[All Fields] | 86 |
| #37 | | SIR-Spheres | | "SIR-Spheres"[All Fields]" | 121 |
| #38 | | SIRSpheres | | "SIRSpheres"[All Fields] | 106 |
| #39 | | #36OR#37  OR#38 | | Microspheres ((SIR-Spheres) OR (SIRSpheres)) OR (TheraSphere) | 182 |
| #40 | | #35OR#39 | | Radioembolization o Yttrium-90 including TheraSphere and SIRSpheres ((((((Yttrium-90) OR (90Y)) OR (90-Y)) OR (Y90)) OR (Y-90)) OR (((((((radioembolization) OR (transarterial radioembolization)) OR (transcatheter arterial radioembolization)) OR (TARE)) OR (Selective internal radiation therapy)) OR (SIRT)) NOT (sirtuins))) OR (((TheraSphere) OR (SIR-Spheres)) OR (SIRSpheres)) | 10,541 |
| #41 | | #19AND#40 | | Population AND Intervention  (((((((hepatocarcinoma) OR (hepatic neoplasms)) OR (primary liver tumour)) OR (primary liver tumors)) OR (Liver metastases)) OR (secondary liver cancer)) OR ((((((((((Carcinoma Hepatocellular) OR (Carcinoma Hepatocelular)) OR (HCC)) OR (intrahepatic cholangiocarcinoma)) OR (Colorectal metastasis)) OR (Colorectal metastases)) OR (Colorectal carcinoma)) OR (Colorectal neoplasms)) OR (colon)) OR (Neuroendocrine tumours))) AND (((((((Yttrium-90) OR (90Y)) OR (90-Y)) OR (Y90)) OR (Y-90)) OR (((((((radioembolization) OR (transarterial radioembolization)) OR (transcatheter arterial radioembolization)) OR (TARE)) OR (Selective internal radiation therapy)) OR (SIRT)) NOT (sirtuins))) OR (((TheraSphere) OR (SIR-Spheres)) OR (SIRSpheres))) | 3,334 |
